# The Many Faces of Purpura: Vancomycin-Induced Leukocytoclastic Vasculitis

**DOI:** 10.1155/2021/9932425

**Published:** 2021-06-04

**Authors:** John A. Zadroga, Vanajakshi Mogulla, Christopher Grant, Djordje Jevtic, Andrew Virata, Igor Dumic

**Affiliations:** ^1^Family Medicine Residency Program, Mayo Clinic Health System, Eau Claire, WI, USA; ^2^Division of Hospital Medicine, Mayo Clinic Health System, Eau Claire, WI, USA; ^3^Mayo Clinic College of Medicine and Science, Rochester, MN, USA; ^4^Medical College of Wisconsin, Milwaukee, WI, USA; ^5^University of Belgrade, School of Medicine, Belgrade, Serbia; ^6^Department of Pathology, Mayo Clinic Health System, Eau Claire, WI, USA

## Abstract

Leukocytoclastic vasculitis is a rare form of immune-mediated vasculitis that might be caused by infections or autoimmune diseases or might be precipitated by specific medications. We describe a 65-year-old patient, who was receiving vancomycin for a methicillin-sensitive *Staphylococcus aureus* permacath infection. Vancomycin was chosen due to medication non-adherence and the patient's desire to receive antimicrobial therapy in conjunction with his scheduled dialysis sessions. The patient's medical history was notable for untreated hepatitis C infection and end-stage renal disease, requiring hemodialysis three times a week. Vancomycin was administered during dialysis sessions. After one week of therapy, the patient developed bilateral lower extremity purpura. Skin biopsy was suggestive of leukocytoclastic vasculitis with an absence of intravascular thrombi. Serum cryoglobulins were negative, making cryoglobulinemia due to HCV infection unlikely. Following cessation of vancomycin therapy, the rash gradually disappeared with scarring in the form of post-purpuric hyperpigmentation. Despite its widespread use, vancomycin is a rare cause of leukocytoclastic vasculitis. Clinicians should keep in mind a wide range of differential diagnosis of bilateral lower extremity purpura as treatment differs depending on its underlying etiology.

## 1. Background

Leukocytoclastic vasculitis (LCV) is an immune complex-mediated disease which affects cutaneous and/or extra cutaneous small blood vessels [1]. Histologically, it is characterized by perivascular neutrophilic infiltration, fibrinoid necrosis, and red blood cell extravasation [1, 2]. Clinically, LCV most commonly manifests as lower extremity palpable purpura [1–3]. It is a relatively rare form of vasculitis with some population-based studies reporting incidence of 45 cases per million per year [4]. Primary (idiopathic) LCV is more common than secondary LCV, which might be caused by infections, neoplastic processes, autoimmune diseases, and medications [1–5]. Drug-induced LCV is most commonly caused by beta-lactam antibiotics, non-steroidal anti-inflammatory drugs, biological agents (infliximab and ustekinumab), and diuretics [3, 6].

Vancomycin is a tricyclic glycopeptide used in its intravenous form for the treatment of Gram-positive bacterial infections, and its oral formulation is used as the first-line treatment of *Clostridioides difficile* colitis. It is widely used for skin, soft tissue, and bloodstream infections caused by methicillin-resistant *Staphylococcus aureus* (MRSA). Common and well-recognized side effects of vancomycin include red man syndrome, ototoxicity, and nephrotoxicity among others [7]. While LCV due to vancomycin has been previously reported, it remains very rare [8–12].

## 2. Case Presentation

A 65-year-old Caucasian man was admitted for 3 days of progressive worsening of a painful rash in his bilateral lower extremities. His medical history was notable for end-stage renal disease secondary to hypertensive nephrosclerosis, chronic untreated hepatitis C virus infection (HCV), coronary artery disease complicated by ischemic cardiomyopathy, and anemia of chronic kidney disease. The patient was non-compliant with his medication management and would intermittently miss hemodialysis appointments as well. One week prior to admission, the patient had started on intravenous vancomycin for a methicillin-sensitive *Staphylococcus aureus* (MSSA) permacath infection. Vancomycin was chosen due to medication non-adherence and the patient's desire to receive antimicrobial therapy in conjunction with his dialysis sessions. He was receiving vancomycin thrice weekly after hemodialysis with the plan to treat the infection for 2 weeks. The patient did not smoke or drink alcohol but had a remote history of intravenous heroin use 15 years prior. He lived in a city apartment and had no recent tick bites, international or domestic travels, or any pets.

**Figure 1:**
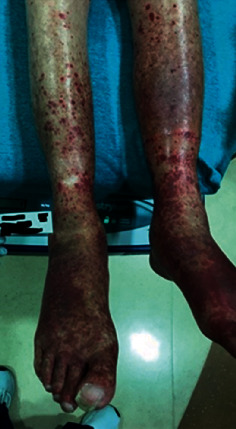
Image of patient's rash, showing non-blanching, purpuric lesions extending from the dorsa of the bilateral feet up to the knees.

The rash initially started as non-painful, bright red spots on his bilateral feet. Gradually, these spots coalesced into a purpuric rash spreading up the bilateral lower extremities and to both arms, sparing the trunk and face (Figure 1). As the rash progressed, it became painful and itchy, which prompted the patient to present for evaluation.

On admission, the patient was afebrile and hemodynamically stable. Initial laboratory studies revealed hyponatremia (Na^+^ = 130 mEq/L), elevated creatinine (6.75 mg/dL), and an estimated glomerular filtration rate (eGFR) < 15 mL/min/1.73 m^2^ consistent with end-stage chronic kidney disease (CKD). He had chronic stable pancytopenia with hemoglobin of 10.2 g/L and platelet count and leukocyte count of 121 and 3.3 × 10^9^/L, respectively. His C-reactive protein (CRP) level was elevated at 27.5 mg/L. Liver function tests and electrolytes were within normal range.

The patient was alert and oriented. His lung sounds were clear and heart sounds were unremarkable, without murmurs or rubs. Neck veins were flat, and oral mucosa was moist without ulceration. Abdomen was non-tender. There was no evidence of small or large joint swelling, pain, or redness. His neurological exam was normal, and in particular, sensation in his lower extremities was intact.

**Figure 2:**
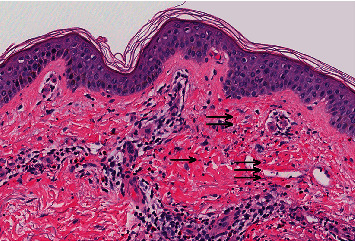
Skin biopsy (H&E stain, 20x magnification) demonstrates dermal inflammation with leukocytoclastic debris (single arrow), extravasated red blood cells (double arrow), and fibrinoid vascular degeneration (triple arrow) consistent with leukocytoclastic vasculitis.

The initial differential diagnosis included cryoglobulinemia secondary to HCV, IgA vasculitis, ANCA-associated vasculitis, and a drug reaction to vancomycin. Further infectious workup included 2 sets of blood cultures, rapid plasma reagin (RPR), HCV RNA, Hepatitis A IgM, Hepatitis B (HBV) surface antigen, and HBV antibody panel. Complement levels (C3 and C4), cryoglobulin, rheumatoid factor, ANCA, ANA, and phospholipid levels were also drawn as part of a workup for inflammatory/autoimmune etiology of the purpura. Vancomycin trough level was noted to be 24.2 mcg/mL. It was discontinued and changed to cefazolin for ongoing treatment of MSSA permacath infection. Skin biopsies were performed (Figure 2).

Serum complement levels were low, with C3 = 60 mg/dL (reference range: 75–175 mg/dL) and C4 = 17 mg/dL (reference range: 14–40 mg/dL). A normal RF of 10 IU/mL (reference range: <15 IU/mL) was also noted, but cryoglobulin levels were within normal limits. Infectious workup returned negative. Serum and urine protein electrophoresis was negative for monoclonal spikes. HCV viral load was 7615 IU/L. The skin biopsy showed perivascular inflammatory-cell infiltrate, comprised predominantly of neutrophils with fibrin deposition within the walls of some small-caliber blood vessels, suggesting disruption with hints of extravasated erythrocytes (Figure 2). There were no thrombi identified to suggest a cryoglobulinemia. Immunofluorescence studies failed to show evidence for IgA vasculitis. Due to normal cryoglobulin levels, RF and C4 complement coupled with the absence of typical symptoms and sign of cryoglobulinemic vasculitis (peripheral neuropathy, arthralgia, fever, etc.) cryofibrinogen levels were not checked, as HCV related cryoglobulinemic vasculitis was deemed unlikely.

Following withdrawal of vancomycin (with subsequent decrease in vancomycin through levels) on hospital day 3, the rash began to subside. The patient completed 2 weeks of therapy with cefazolin and obtained near-complete resolution of the rash one month after the presentation. As the rash started improving before definitive diagnosis was made, steroids were not given.

## 3. Discussion

LCV manifests as areas of transmural infiltration of neutrophils and disruption of the blood vessel structure. After degranulation, neutrophils undergo death, hence the term leukocytoclasia. Skin biopsy, in addition to neutrophil infiltration around the vessel wall may demonstrate other findings such as granulocytic debris, extravasated erythrocytes, and endothelial swelling. Biopsy is site and time dependent. If lesions are biopsied within 12 hours of the rash onset or greater than 48 hours, largely lymphocytic infiltrate is present and leukocytoclasis may be absent [1, 13, 14]. The skin biopsy in our patient was consistent with LCV and demonstrated the absence of thrombi, which made cryoglobulinemia-associated LCV less likely. Immunofluorescence did not show evidence of IgA deposits. Infectious workup was negative making infectious LCV less likely. Bilateral distribution of the rash and lack of fever argued against cellulitis. Taking into account all these data, the diagnosis of LCV secondary to vancomycin was entertained. Our hypothesis is further supported by the fact that the rash resolved spontaneously following withdrawal of vancomycin and negative extensive workup for infectious and autoimmune conditions.

The most common dermatological manifestation of LCV is non-blanching palpable purpura; however, exclusive erythema, maculopapular or petechial rash, and nodules have been reported as well [3]. The most important differential diagnoses of palpable purpura include cryoglobulinemia, IgA vasculitis, Henoch–Schonlein purpura (HSP), polyarthritis nodosa due to hepatitis B, calciphylaxis, warfarin-induced skin necrosis, and thrombotic microangiopathy (TMA) [15]. HSP usually occurs in children following upper respiratory infection, and the extent of the skin changes is less that what was observed in our patient. There were no schistocytes on peripheral blood smear to suggest TMA, and our patient was not on warfarin therapy; Calciphylaxis was ruled out by skin biopsy.

The exact sensitivity of tests for cryoglobulins is unknown and, to the best of our knowledge, it has not been evaluated in prospective studies. Diagnosis of HCV-related cryoglobulinemic vasculitis is based on the combination of clinical manifestation (purpura, arthralgia, fever, peripheral neuropathy, glomerulonephritis, etc.), positive HCV RNA, elevation in RF, positive cryoglobulins, and decrease in C4 complement level. While our patient had purpura and an elevated HCV viral load, he did not meet the other aforementioned criteria. While theoretically the presence of falsely negative cryoglobulins in the setting of HCV vasculitis is possible, it is more common to have elevated cryoglobulin (in particular mixed type II and III) in the setting of HCV even without purpura [16–18]. Furthermore, HCV-related cryoglobulinemic vasculitis responds well to anti-HCV therapy. As our patient improved without specific antiviral therapy or immunosuppressive medications, one can argue that it ruled out HCV-related vasculitis. In the absence of antiviral and immunosuppressive therapy, cryoglobulinemic HCV-related vasculitis would progress rather than recede.

A PubMed search was performed in January 2021 to explore literature published within the past 10 years including the key words “leukocytoclastic vasculitis” and “vancomycin.” This yielded only 4 previously published case reports and 1 letter. These cases also demonstrated onset of rash shortly (within days) after initiation of vancomycin therapy, similar to what was observed in our patient. Distribution of the rash in these cases primarily involved the upper/lower extremities with variable involvement of the abdomen/trunk, and convalescence of the rash was noted shortly (within weeks) after cessation of vancomycin treatment. Among the 5 reported patients, only one was noted to have ESRD and he was also male.

Treatment of LVC depends on underlying etiology. In cases of drug-induced LCV, drug withdrawal is sometimes enough to achieve LCV resolution [19] as demonstrated in the current case. However, in cases where the skin lesions are extensive or progress despite withdrawal of the offending medication, use of immunosuppressive therapy has been demonstrated to be effective [3]. Steroids are most frequently used, usually at the dose of 1 mg/kg with a taper. In the majority of cases, lesions resolve without scarring; however, as seen in our patient, post-purpuric hyperpigmentation can be a long-term esthetic sequela. Colchicine has been used successfully in case reports of steroid refractory LCV [20].

## Data Availability

Data are available from the corresponding author upon request.
